# Load Resistance Optimization of a Magnetically Coupled Two-Degree-of-Freedom Bistable Energy Harvester Considering Third-Harmonic Distortion in Forced Oscillation

**DOI:** 10.3390/s21082668

**Published:** 2021-04-10

**Authors:** Jinhong Noh, Pilkee Kim, Yong-Jin Yoon

**Affiliations:** 1 Department of Mechanical Engineering, Korea Advanced Institute of Science and Technology, Daejeon 34141, Korea; jinhongnoh@kaist.ac.kr; 2 School of Mechanical Design Engineering, College of Engineering, Jeonbuk National University, 567 Baekje-daero, Deokjin-gu, Jeonju-si 54896, Korea; 3 Eco-Friendly Machine Parts Design Research Center, Jeonbuk National University, 567 Baekje-daero, Deokjin-gu, Jeonju-si 54896, Korea; 4 School of Mechanical and Aerospace Engineering, Nanyang Technological University, Singapore 639798, Singapore

**Keywords:** bistable energy harvester, optimal resistance, harmonic distortion, broadband performance

## Abstract

In this study, the external load resistance of a magnetically coupled two-degree-of-freedom bistable energy harvester (2-DOF MCBEH) was optimized to maximize the harvested power output, considering the third-harmonic distortion in forced response. First, the nonlinear dynamic analysis was performed to investigate the characteristics of the large-amplitude interwell motions of the 2-DOF MCBEH. From the analysis results, it was found that the third-harmonic distortion occurs in the interwell motion of the 2-DOF MCBEH system due to the nonlinear magnetic coupling between the beams. Thus, in this study, the third-harmonic distortion was considered in the optimization process of the external load resistance of the 2-DOF MCBEH, which is different from the process of conventional impedance matching techniques suitable for linear systems. The optimal load resistances were estimated for harmonic and swept-sine excitations by using the proposed method, and all the results of the power outputs were in excellent agreements with the numerically optimized results. Furthermore, the associated power outputs were compared with the power outputs obtained by using the conventional impedance matching technique. The results of the power outputs are discussed in terms of the improvement in energy harvesting performance.

## 1. Introduction

### 1.1. Introduction to Bistable Energy Harvesters

Energy harvesting, which draws useful electrical energy from environmental energy sources [1,2,3,4,5,6,7], remains an active research area for various promising applications such as smart environments and wireless sensor networks [8]. Energy harvesting has been considered as a possible alternative powering technology to chemical batteries [9]. When wireless sensor networks are powered by energy harvesting technology, maintenance cost for battery replacement in places like buildings, bridges, or tires would be unnecessary [10,11]. In addition, many researchers have been trying to apply energy harvesting technology to a variety of emerging applications, such as electrochemical cycles [12], energy-autonomous electronic skins [13], or wireless power transfer for implantable devices [14].

**Figure 1:**
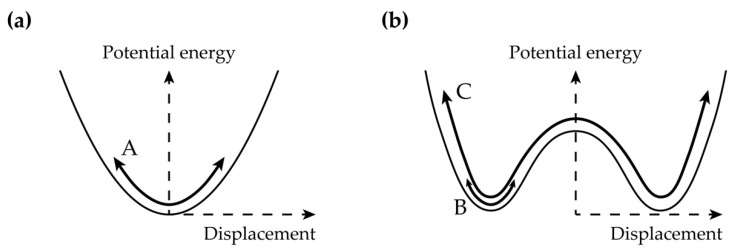
Schematics of the potential energy functions of (**a**) the monostable system and (**b**) the bistable system. In (**a**), the monostable system has only a single-well oscillation plotted by A. In contrast, in (**b**), the bistable system has not only the intrawell motion but also the interwell motion, depicted by B and C, respectively. An attractor for complex chaotic motion might coexist with regular behavior, but the chaotic motion is not investigated in this research.

Nonlinear vibration-based energy harvesters (VEHs) have gained intensive attention over the past decade for broadband energy harvesting applications [15,16]. A bistable energy harvester (BEH) is the fundamental design of a nonlinear VEH [17]. The energy harvester in this study utilizes the bistable configuration in which the potential energy, as a function of displacement, has two local minima. Figure 1a shows the behavior of the monostable system, which has the single-well potential energy function. Path A in Figure 1a represents the single-well oscillation. Figure 1b depicts the double-well potential energy of the bistable system. The intrawell, interwell, and chaotic motions of the bistable system appear, depending on the initial conditions and the excitation [18]. When the intrawell motion appears, the system vibrates along path B in Figure 1b. Because the oscillation occurs within one of the two potential wells, the amplitude of the oscillation is insufficient for energy harvesting [19]. In contrast, bistable energy harvesters are capable of extracting adequate power when oscillating along path C in Figure 1b. The system vibrates across the wells when the state overcomes the saddle barrier, which is the local maximum point between the wells. The output power harvested from this large-amplitude interwell motion is bigger than that from the intrawell motion [20]. Thus, the main research effort in this study was focused on the dynamical behavior in interwell motion in order to optimize the energy harvesting performance.

### 1.2. Contribution of This Study

**Figure 2:**
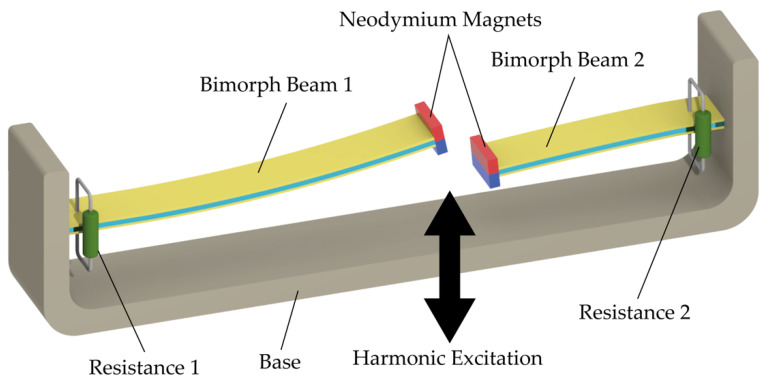
Three-dimensional view of the two-degree-of-freedom bistable energy harvester (2-DOF MCBEH). A longer beam located on the left is the bimorph beam 1, and a shorter beam located on the right is the bimorph beam 2. The identical permanent magnets are attached to the tips of the beams. The base, considered as a rigid body, excites the beams. The harmonic motion of the base is confined to upward and downward movement. The energy harvester scavenges this vibrational energy to extract electrical power.

Figure 2 shows the schematic of a magnetically coupled two-degree-of-freedom bistable energy harvester (2-DOF MCBEH) considered in this study. As shown in Figure 2, the two piezoelectric bimorph beams are cantilevered into the base structure at the same height on the opposite side. The two identical neodymium magnets are attached to the free ends of the cantilevered beams, so that they are forced oppositely by repulsive magnetic forces. The piezoelectric layers of the bimorph beams are poled in the thickness direction and connected to the external load resistances in series. Such a 2-DOF MCBEH is known to have superior broadband performance to conventional 1-DOF BEHs [21]. In this study, the nonlinear dynamical behaviors of the 2-DOF MCBEH were investigated. From the results of the dynamic analyses, the third-harmonic distortion in the interwell motion of the 2-DOF MCBEH was observed and considered important in the optimization process of the external load resistances to maximize the energy harvesting performance.

Impedance matching is the most well-known technique for the load resistance optimization of VEHs [22]. The main object of this technique is to obtain the equivalent impedance of the mechanical part with an equivalent circuit model. For example, Song et al. measured the internal impedance of the VEH at driving frequency and matched load resistance to this measured impedance [23]. Liu et al. matched the resistance of the bistable energy harvester at linear resonance frequency to optimize performance [24]. However, the use of this impedance-matching technique for bistable energy harvesters is based on the oversimplification that only the fundamental frequency component of response is considered in the load resistance optimization [25]. In previous works [26,27], the load resistance optimizations of the 1-DOF bistable piezoelectric and electromagnetic energy harvesters were performed for primary harmonic and subharmonic interwell motions. It was demonstrated that the optimal load resistance strongly depended on the oscillation frequency of the interwell motion rather than on the forcing frequency of excitation. Although harmonic distortions in the interwell motions of the 1-DOF systems were not significant [26,27], it can be readily deduced that high-order frequency components are important in the optimization of the external load resistance. Thus, the impedance matching technique is possibly undesirable and inappropriate for the load resistance optimization of the 2-DOF MCBEH when certain high-order harmonics are significant in response (e.g., the third-harmonic distortion of beam 2 presented later in this study).

The nonlinear dynamic analyses and load resistance optimization were performed in this study to maximize the energy harvesting performance of the 2-DOF MCBEH. First, frequency response analysis was conducted to find the operating frequency range of the 2-DOF MCBEH, where the large-amplitude interwell motion occurs. Second, to investigate the effects of high-order harmonics on the dynamic response, the interwell motions were analyzed in the frequency domain by the fast Fourier transform. It was found that for beam 2, the third-order harmonic component as well as the fundamental harmonic component plays an important role in the interwell oscillation of the 2-DOF MCBEH, which leads to the third-harmonic distortion. Therefore, in this study, an optimization process for the load resistance was established with consideration of the third-harmonic distortion. The optimal load resistances estimated by the proposed optimization method were compared with those evaluated by the numerical optimization process and the conventional impedance matching technique. The results of this study imply that the proposed optimization process, in which the third-harmonic distortion is considered, can be extended to various fields [28,29].

## 2. Model Description

### 2.1. Geometric Dimensions and Material Properties

**Figure 3:**
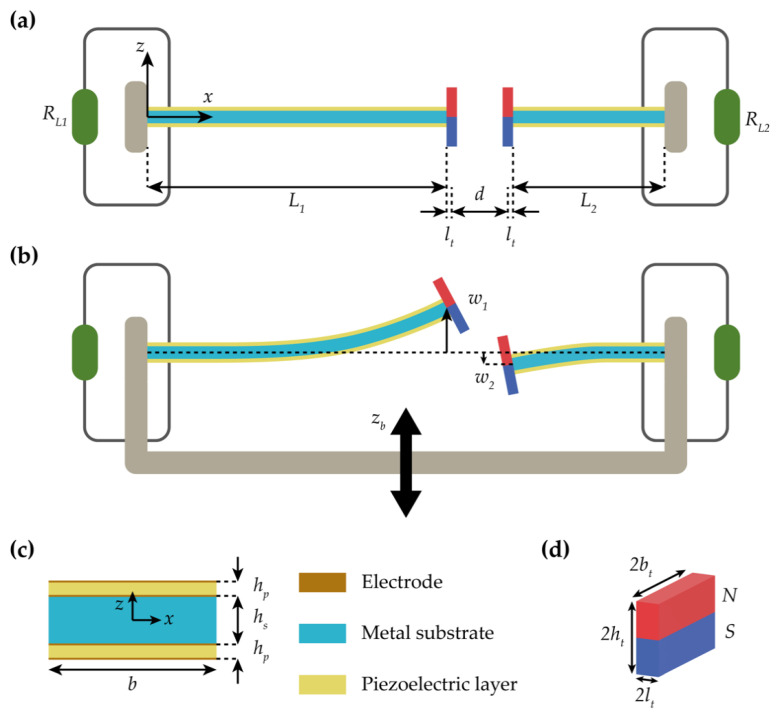
Geometric configurations and a schematic of motion of the 2-DOF MCBEH. Notations for geometric dimensions and coordinate system are depicted in (**a**). (**a**) shows that the external resistances are connected to the bimorph beam in series. (**b**) is a schematic of motion of the 2-DOF MCBEH in the *x*-*z* plane. The system is excited by the harmonic base displacement. The sign conventions of the tip deflections of the beams are opposite to each other. The out-of-phase motion of the beams is depicted in (**b**). (**c**) gives the magnified cross-sectional view of the bimorph beams. Each beam has the same dimensions except for the length. The thickness of the electrodes is neglected because it is too thin. The geometry of two identical neodymium magnets is shown in (**d**). The center of the magnet is considered to coincide with the centroid of the bimorph beam.

Figure 3a shows the geometric configurations of the 2-DOF MCBEH. A coordinate system is fixed at the clamped end of the bimorph beam 1. The *x*-axis is in the longitudinal direction of the beam along the neutral surface, and the *z*-axis is in the thickness direction. The gravitational force is assumed to act in the *y*-direction. In Figure 3a, $L_{i}$ and $R_{Li}$ are the length of the beam and the external load resistance, respectively, where the symbol *i* (=1 or 2) in the subscript indicates beam 1 or 2, $l_{t}$ is the half length of the permanent magnet, and *d* is the separation distance between the centers of mass of two magnets when the beams are undeformed. Figure 3b shows a schematic of the motion of the 2-DOF MCBEH. In this figure, $z_{b}$ is the displacement of the base structure excited harmonically in the *z*-direction and $w_{i}$ (*i* = 1 or 2) is the transverse deflection of the tip. The sign of $w_{1}$ is positive for upward motion and opposite to the sign of $w_{2}$, as depicted in Figure 3b. Figure 3c shows a cross-sectional view of the bimorph beam. In this figure, *b* is the width of the beam, $h_{s}$ and $h_{p}$ are the thicknesses of the metal substrate and piezoelectric layers, respectively, and the thickness of the electrode is neglected. The dimensions of the permanent magnet are depicted in Figure 3d, where $2h_{t}$ and $2b_{t}$ are the thickness and width of the two identical neodymium magnets, respectively. The values of the geometric dimensions and material properties used in this study are listed in Table 1.

### 2.2. Mathematical Model of the 2-DOF MCBEH

For the curvatures of the beams, the Euler–Bernoulli beam theory for perfectly bonded composite structures was employed. For the constitutive relation, the linear elastic material model was utilized. The permanent magnets (or tip masses) were approximated to be perfectly constrained at the tips of each beam and have the same rotational angle as the that of the tips. The magnetic charge model was applied to describe the magnetic repulsive force acting on the tip in free space. Using Hamilton’s principle, the governing field equations were derived and, subsequently, the modal analysis was performed to obtain the linear natural frequencies and mode shapes [30]. After imposing the single mode approximation and discretization assumption on the governing equations in modal coordinates, the oscillator model of the 2-DOF MCBEH can be derived as follows [31]:

(1) $${\ddot{w}}_{i}+2\zeta_{i}\omega_{i}{\dot{w}}_{i}+\omega_{i}^{2}w_{i}-\beta_{i}V_{i}-F_{mi}=-\alpha_{i}{\ddot{z}}_{b},\;i=1,2,$$

(2) $${\dot{V}}_{i}+\eta_{i}V_{i}+\gamma_{i}{\dot{w}}_{i}=0,\;i=1,2,$$

where $V_{i}$ is the voltage response, $\zeta_{i}$ is the damping ratio, $\omega_{i}$ is the linear natural frequency, $\beta_{i}$ is the modal electromechanical coupling coefficient for motion, $F_{mi}$ is the modal magnetic force, $\alpha_{i}$ is the modal excitation coefficient, $\eta_{i}$ is the modal resistance, and $\gamma_{i}$ is the modal electromechanical coupling coefficient for circuit. Herein, the modal resistance and base excitation are expressed by

(3) $$\eta_{i}=\frac{2}{C_{pi}R_{Li}},$$

(4) $${\ddot{z}}_{b}=F_{b}\cos\left(\Omega t\right)$$

where $C_{pi}$ is the equivalent capacitance for the piezoelectric layer, and $F_{b}$ and Ω are the amplitude and frequency of the base excitation, respectively.

For the geometric dimensions and material properties listed in Table 1, the values of the parameters were obtained as follows: $\omega_{1}=1.5461\times 10^{2}$ rad/s, $\omega_{2}=3.4785\times 10^{2}$ rad/s, $\beta_{1}=-1.6474\times 10^{-3}$ C/kg·m, $\beta_{2}=-3.0738\times 10^{-3}$ C/kg·m, $\alpha_{1}=1.1597$, $\alpha_{2}=-1.0870$, $\eta_{1}=1.3141\times 10^{3}$/s, $\eta_{2}=2.1802\times 10^{3}$/s, $\gamma_{1}=-3.2167\times 10^{3}$ V/m, and $\gamma_{2}=-8.9713\times 10^{3}$ V/m. In addition, both of the damping ratios, $\zeta_{1}$ and $\zeta_{2}$, were chosen as 0.007.

## 3. Methods: Dynamic Simulation and Optimization

### 3.1. Dynamical Characterization Method

For numerical dynamic simulation, the second-order ordinary differential equations (ODEs) given by Equation (1,2) were converted by introducing the following state vector,

(5) $$\vec{x}={\left[\begin{array}{cccccc} x_{1} & x_{2} & x_{3} & x_{4} & x_{5} & x_{6} \end{array}\right]}^{T}={\left[\begin{array}{cccccc} {\dot{w}}_{1} & w_{1} & V_{1} & {\dot{w}}_{2} & w_{2} & V_{2} \end{array}\right]}^{T},$$

into a system of the first-order ODEs:

(6) $$\dot{\vec{x}}=\left(\begin{array}{c} -2\zeta_{1}\omega_{1}x_{1}-\omega_{1}^{2}x_{2}+\beta_{1}x_{3}+F_{m1}-\alpha_{1}{\ddot{z}}_{b}\\ x_{1}\\ -\gamma_{1}x_{1}-\eta_{1}x_{3}\\ -2\zeta_{2}\omega_{2}x_{4}-\omega_{2}^{2}x_{5}+\beta_{2}x_{6}+F_{m2}-\alpha_{2}{\ddot{z}}_{b}\\ x_{4}\\ -\gamma_{2}x_{4}-\eta_{2}x_{6} \end{array}\right).$$

The direct numerical integration of Equation (6) was performed by implementing the Runge–Kutta method. The frequency responses for the beam deflections and output voltages were evaluated by means of sine sweep technique with zero initial conditions. To analyze the nonlinear behaviors of the 2-DOF MCBEH, the excitation frequency was increased from 11.9 Hz to 17.5 Hz, where the third-harmonic distortion in the interwell motion of beam 2 occurs. The amplitude of the base excitation was set to be 6 m/s^2^.

### 3.2. Analytical Optimization Method for Load Resistance

Frequency-domain analysis was performed by the fast Fourier transform of the time responses obtained by the numerical simulation. Based on the frequency-domain results, the tip deflection and voltage across the external load resistance can be expressed by

(7) $$w_{i}=\overset{\infty }{\underset{n=1}{\sum }}\left\{W_{in}e^{in\Omega t}+W_{in}^{*}e^{-in\Omega t}\right\},$$

(8) $$V_{i}=\overset{\infty }{\underset{n=1}{\sum }}\left\{\Psi_{in}e^{in\Omega t}+\Psi_{in}^{*}e^{-in\Omega t}\right\}$$

where *n* indicates the order of the harmonic term and Ω is the angular excitation frequency.

Substituting Equations (7) and (8) into Equation (2) and performing mathematical manipulation lead to

(9) $$\Psi_{in}=\frac{-in\Omega \gamma_{i}}{in\Omega +\eta_{i}}W_{in},$$

and the resulting root mean square (RMS) output power becomes

(10) $$P_{RMSi}=\frac{V_{RMSi}^{2}}{R_{Li}}=2\overset{\infty }{\underset{n=1}{\sum }}\frac{{\left(n\Omega \gamma_{i}\right)}^{2}}{R_{Li}\left\{{\left(n\Omega \right)}^{2}+\eta_{i}^{2}\right\}}{\left|W_{in}\right|}^{2}.$$

An equation for maximizing the RMS power, $\partial P_{RMSi}/\partial R_{Li}|{}_{R_{Li}=R_{opti}}=0$, where $R_{opt}$ represents the optimal load resistance, takes the form:

(11) $$\overset{\infty }{\underset{n=1}{\sum }}\frac{n^{2}\left\{\eta_{opti}^{2}-{\left(n\Omega \right)}^{2}\right\}{\left|W_{in}\right|}^{2}}{{\left\{\eta_{opti}^{2}+{\left(n\Omega \right)}^{2}\right\}}^{2}}=0.$$

Solving Equation (11) for $\eta_{opti}$ gives the optimal load resistance as follows:

(12) $$R_{opti}=\frac{2}{\eta_{opti}C_{pi}}.$$

## 4. Results and Discussion

### 4.1. Frequency Response near the First Primary Resonance

**Figure 4:**
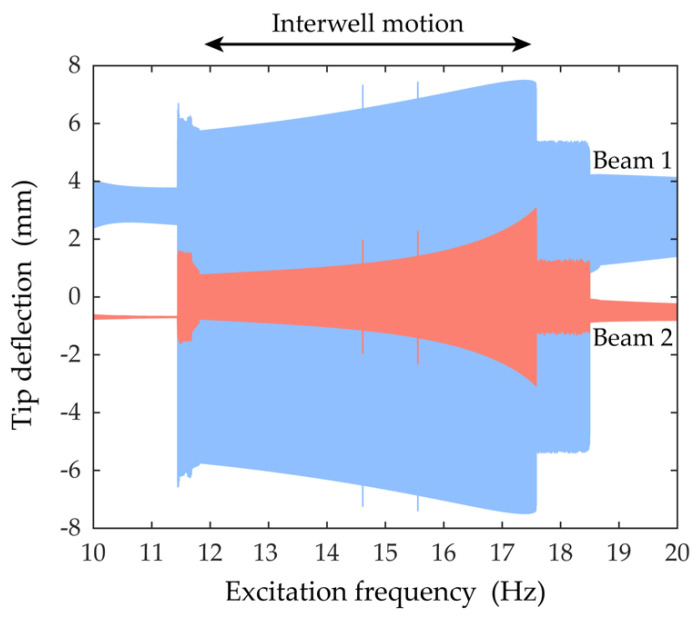
Swept sine response is shown. To identify the region in which attractors for interwell motion exist, the forward-sweep response is overlaid with the backward-sweep response. The marked region, from 11.9 to 17.5 Hz, above the graph indicates where the regular attractors for interwell motion exist. The amplitude of the excitation was set to be 6 m/s^2^.

Figure 4 shows the frequency responses of the piezoelectric bimorph beams of the 2-DOF MCBEH near the first primary resonance under the harmonic base excitation, of which the amplitude is set to be 6 m/s^2^. The bifurcation structure of the 2-DOF MCBEH has been already studied [31]. Because the amplitude of the excitation is strong enough to surmount the potential saddle barrier, the potential well escape phenomena occur and lead to the large-amplitude interwell motion, as shown in Figure 4. For the forward-sweep response, a discontinuous change in the amplitude is observed when the excitation frequency reaches 14.6 Hz. This jump phenomenon is initiated by the saddle-node bifurcation of the intrawell motion, launching the periodic interwell motion. Subsequently, the amplitude of the interwell motion persists in growing until the excitation frequency becomes 17.6 Hz, at which point the motion jumps down onto the cross-well chaotic motion by the saddle-node bifurcation of the interwell motion. For the backward-sweep response, the T-periodic intrawell motion is transformed into the 2T-periodic oscillation by the period-doubling bifurcation, when the excitation frequency is 18.7 Hz. Afterward, the period-doubling cascade is developed, and the cross-well chaotic motion begins when the excitation frequency is 18.4 Hz. At the excitation frequency of 15.6 Hz, the boundary crisis occurs and leads to the unique large-amplitude interwell motion. The periodic interwell motion perishes when reaching the Neimark–Sacker bifurcation, and the unstable motion becomes stabilized when jumping down onto the solution branch of the periodic intrawell motion. Although the multiple responses (e.g., intrawell, interwell, and chaotic motions) can coexist at the same excitation frequency, the regular interwell motion is our main interest, so it was investigated and its frequency range was identified. The marked region above the graph in Figure 4, from 11.9 to 17.5 Hz, indicates where the interwell motion can be found. This frequency interval between 11.9 to 17.5 Hz was chosen to analyze nonlinear dynamic behavior in detail.

### 4.2. Third-Harmonic Distortion in Interwell Motion

**Figure 5:**
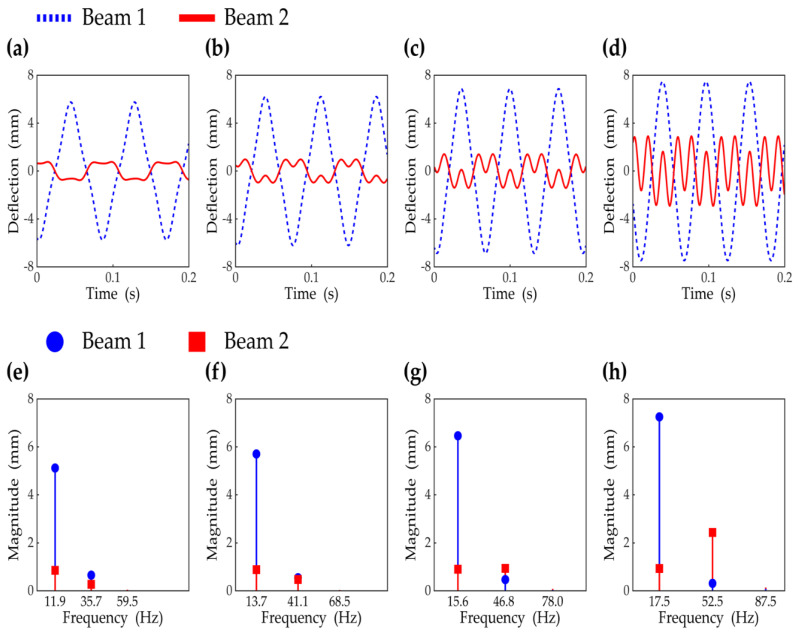
The steady-state interwell responses of the two piezoelectric bimorph beams are shown. (**a**–**d**) are the time responses obtained when the excitation frequencies are 11.9 Hz, 13.7 Hz, 15.6 Hz, and 17.5 Hz, respectively, which belong to the frequency region of the first primary resonance. The amplitude of the excitation is 6 m/s^2^. The magnitudes of the harmonic components in the time responses (**a**–**d**) are plotted in (**e**–**h**). It is observed that the magnitudes of the fifth harmonic terms are negligible for all the cases (**e**–**h**).

The steady-state responses in the region of the first primary resonance are presented in the time and frequency domains in Figure 5. The interwell motions were obtained at the excitation frequencies of 11.9 Hz, 13.7 Hz, 15.6 Hz, and 17.5 Hz and were compared to each other to demonstrate changes in the responses of the 2-DOF MCBEH as the excitation frequency increased. As shown in Figure 5a–d, the tip deflection of beam 1 is amplified at a higher excitation frequency; however, the form of the oscillation stays unchanged. As observed in Figure 5a, when the excitation frequency is low, beam 2 oscillates almost out-of-phase with beam 1. However, for beam 2, the partial in-phase swing motion becomes more notable as the excitation frequency approaches 17.5 Hz. Figure 5e–h shows the results of applying the fast Fourier transform to Figure 5a–d. For both beams, the fundamental and third-order harmonic components are observed and compared. For beam 1, the third-harmonic component is always much smaller than the fundamental-harmonic component and tends to be more suppressed as the excitation frequency increases. This means that the distortion of the primary harmonic response produced by the third-harmonic component is negligible. On the other hand, for beam 2, the effect of the third-harmonic component (and thus the third-harmonic distortion) is more significant with the increase of the excitation frequency. The third-harmonic component in the response of beam 2 is relatively small at the lowest excitation frequency (Figure 5e), but it continues to increase with the excitation frequency (Figure 5f), and finally, it becomes larger than the fundamental-harmonic component (Figure 5g–h). Figure 5d,h clearly demonstrates that for beam 2, the third-harmonic distortion in the response is remarkable, while negligible for beam 1. The rapid growth in the third-harmonic distortion of beam 2 results in the amplification of the in-phase swing of beam 2 in Figure 5a–d.

According to the results of the fast Fourier transform, total harmonic distortion denoted by $K_{i}$ reduces to the third-harmonic distortion as follows:

(13) $$K_{i}≜\frac{\sqrt{W_{i2}^{2}+W_{i3}^{2}+W_{i4}^{2}+\cdots }}{\left|W_{i1}\right|}\approx \frac{\left|W_{i3}\right|}{\left|W_{i1}\right|}.$$

**Figure 6:**
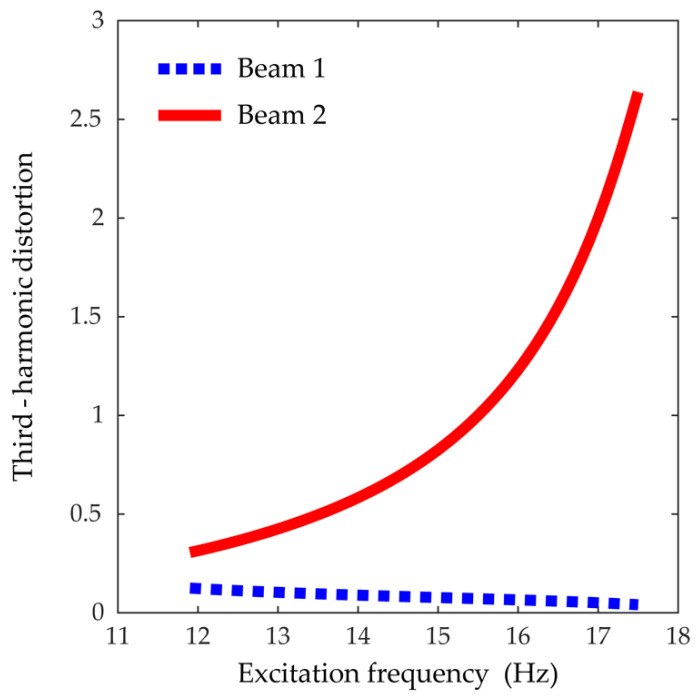
The quantitative third-harmonic distortions, the ratios of the magnitude of the third sinusoidal component to the magnitude of the fundamental component, of the steady-state response in interwell motion. In the calculations, the amplitude of the excitation was set to be 6 m/s^2^.

Thus, the third-harmonic distortion measures the relative contribution of the third-harmonic component to the fundamental-harmonic component towards the interwell motion. Referring to Figure 6, as the excitation frequency approaches 17.5 Hz, the quantitative third-harmonic distortion of beam 1, evaluated by using Equation (13), tends to slightly decrease, and its absolute value remains small in the entire frequency band; on the other hand, the third-harmonic distortion of beam 2 soars dramatically, which means that the third-harmonic distortion has a significant effect on the nonlinear interwell motion of the 2-DOF MCBEH.

### 4.3. Optimization of the External Load Resistance of the 2-DOF MCBEH

The load resistance optimization is proposed in this section, considering third-harmonic distortion. As shown in the previous section, the interwell motion of beam 2 can be assumed to have the fundamental- and third-harmonic components, and accordingly the optimization problem given by Equation (11) reduces to the form:

(14) $$\overset{}{\underset{n=1,3}{\sum }}\frac{n^{2}\left\{\eta_{opti}^{2}-{\left(n\Omega \right)}^{2}\right\}{\left|W_{in}\right|}^{2}}{{\left\{\eta_{opti}^{2}+{\left(n\Omega \right)}^{2}\right\}}^{2}}=0.$$

Rearranging Equation (14) gives a polynomial equation in the following vector form:

(15) $${\left(\begin{array}{c} N^{2}K_{i}^{2}+1\\ \left(-N^{4}K_{i}^{2}+2N^{2}K_{i}^{2}+2N^{2}-1\right)\Omega^{2}\\ \left(-2N^{4}K_{i}^{2}+N^{4}+N^{2}K_{i}^{2}-2N^{2}\right)\Omega^{4}\\ \left(-N^{4}K_{i}^{2}-N^{4}\right)\Omega^{6} \end{array}\right)}^{T}\left(\begin{array}{c} \eta_{opti}^{6}\\ \eta_{opti}^{4}\\ \eta_{opti}^{2}\\ 1 \end{array}\right)=0.$$

The optimal load resistance $R_{opti}$ considering the third-harmonic distortion can be obtained by solving Equation (15).

For the case of the conventional impedance matching technique commonly used for linear systems, only the fundamental-harmonic component of the response is considered in the optimization process. In this case, the optimization problem further reduces to $\eta_{opti}^{2}-\Omega^{2}=0$ by taking n=1 only in Equation (14), and the matched load resistance $R_{mat}$ can be obtained in the closed form:

(16) $$R_{opti}=R_{mati}=\frac{2}{\Omega C_{pi}}\;\mathrm{for}\;n=1\;\mathrm{only}.$$

**Figure 7:**
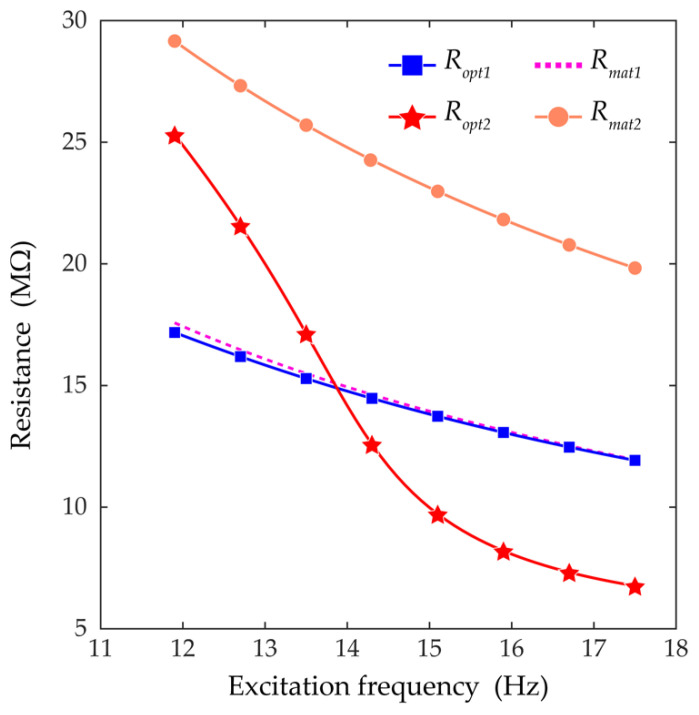
The optimal load resistances and the matched load resistances at each excitation frequency are plotted. *R_opt1_* and *R_opt2_* are the optimal resistances for beam 1 and beam 2, respectively. *R_mat1_* and *R_mat2_* are the matched resistances for beam 1 and beam 2, respectively. The resistances are calculated at the excitation frequencies from 11.9 to 17.5 Hz with increments of 0.1 Hz. The third-harmonic distortions for calculating the optimal resistances were obtained when the excitation amplitude was 6 m/s^2^.

Figure 7 shows a comparison of the optimal load resistance (obtained by the proposed method) to the matched load resistance (obtained by the conventional impedance matching method). For beam 1, because the third-harmonic distortion was negligible, small discrepancies between the optimal resistances and the matched resistances are observed. In contrast, for beam 2, because the third-harmonic distortion was relatively small at a low frequency and notably large at a high frequency, the differences between the optimal resistances and the matched resistances become more significant as the excitation frequency increases. This suggests that for beam 2, the third-harmonic distortion plays a prominent role in the optimization of the load resistance and the proposed optimization method, based on Equation (15), is likely to result in more improved output power when compared with that of the conventional impedance matching technique.

**Figure 8:**
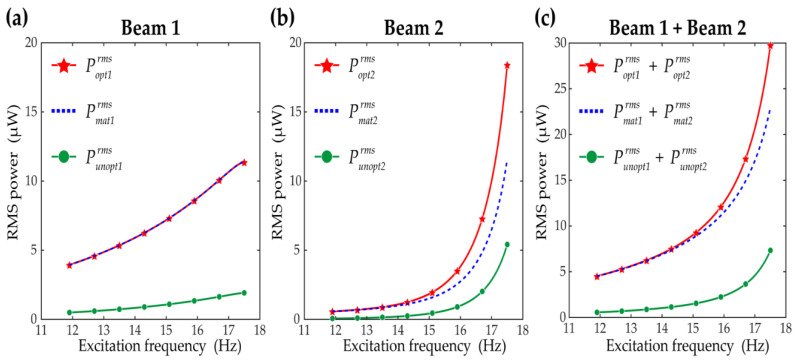
Root mean square (RMS) power output results of (**a**) beam 1 and (**b**) beam 2, and (**c**) total RMS output power results of the two beams. The red lines with the stars are the results obtained with the optimal load resistances considering the third-harmonic distortion. The blue dotted lines are the results obtained by applying the impedance matching technique considering the fundamental component only. The green lines with the circles are the RMS power output evaluated with the resistance of 1 MΩ without load resistance optimization. The excitation frequencies are increased from 11.9 Hz to 17.5 Hz with increments of 0.1 Hz, and the base acceleration was set to be 6 m/s^2^.

Figure 8 shows how much RMS power output is improved with the optimal load resistance obtained by the proposed method, compared to the RMS power output with the matched resistances. In addition, 1 MΩ was chosen as an unoptimized resistance value, and the RMS power results calculated with the unoptimized resistance are shown for comparisons. In Figure 8, $P_{opt}^{rms}$, $P_{mat}^{rms}$, and $P_{unopt}^{rms}$ denote the RMS power outputs evaluated with the optimal, matched, and unoptimized resistances, respectively. The subscripts 1 and 2 indicate the results for beam 1 and beam 2, respectively. The RMS power output produced by beam 1 is shown in Figure 8a. For beam 1, the RMS power graphs with the optimized and matched resistances overlap because as shown in Figure 7, those resistances had ineffective differences. At the highest excitation frequency, 17.5 Hz, both the RMS power results with the optimized and matched resistances are improved by 5.93 times when compared to the RMS power with the unoptimized resistance. On the other hand, for beam 2, the RMS power output is more improved with the optimal resistances than with the matched resistances, as shown in Figure 8b. This is more obvious as the excitation frequency increases owing to the differences between the optimal and matched resistances in Figure 7. Particularly, at the excitation frequency of 17.5 Hz, the RMS power output with the optimal resistances is enhanced by 3.40 times relative to the RMS power output with the unoptimized resistances, whereas the matched resistances achieve a 2.13 times enhancement. The total RMS power output produced by both of the two piezoelectric beams are shown in Figure 8c. Because for beam 1 the optimized and matched resistances showed no difference in the RMS power outputs, the difference between the total power output with the optimized resistances and the total power output with the matched resistances originates mainly from beam 2. Resultantly, at the excitation frequency of 17.5 Hz, the total RMS power outputs with the optimized and matched resistances are improved by 4.06 times and 3.13 times, respectively, relative to the total RMS power with the unoptimized resistance.

### 4.4. Improvements in Broadband Performance

Enhancing broadband performance is of importance when designing VEHs [32]. Herein, for quantitative comparisons of broadband performance, average RMS power, denoted by $E_{i}$, is introduced. The average RMS power is evaluated by dividing the area under the RMS power curve by the excitation frequency range in which interwell motion occurs. The average RMS power obtained when the resistances are optimized at 17.5 Hz is denoted by $E_{opt}$. Likewise, $E_{mat}$ represents the average RMS power obtained when the resistances are matched at 17.5 Hz. In addition, for comparison, $E_{unopt}$ denotes the average RMS power obtained with the unoptimized resistances of 1 MΩ. The area under an RMS power curve is numerically integrated by utilizing Simpson’s 1/3 rule. To investigate the broadband performance of the 2-DOF MCBEH with respect to changes of load resistances, the values of the average RMS power were evaluated with variations in the load resistances of both the beams from 1 to 35 MΩ with increments of 0.5 MΩ.

**Figure 9:**
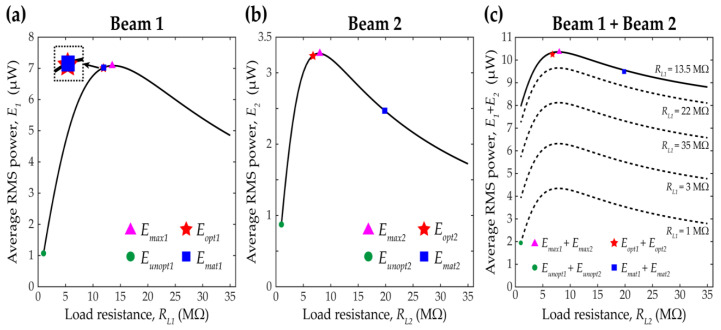
Average RMS power results for beam 1 and beam 2 are shown in (**a**,**b**), respectively, with changing external load resistances. (**c**) shows the summation of (**a**,**b**). Each resistance varies from 1 MΩ to 35 MΩ with increments of 0.5 MΩ for the simulations. The average RMS power of beam 1 is assumed to be independent of the load resistance of beam 2, and vice versa. The load resistance of beam 2 is set to be 8 MΩ for (**a**), and the load resistance of beam 1 is chosen as 13.5 MΩ for (**b**). The magenta triangle marker denotes the maximum average RMS power. The red star marker indicates the average RMS power obtained when the resistance is optimized at the excitation frequency of 17.5 Hz; on the other hand, the blue square is obtained with the matched resistances at the same excitation frequency. For comparisons, the average RMS power with the resistances of 1 MΩ is denoted by the green circle marker.

Figure 9 depicts the simulation results for the broadband performance estimated by the average RMS power. First of all, it is noted that the average RMS power of beam 1 is practically independent of the variations of the resistance connected to beam 2, and vice versa. In this regard, for simplification, Figure 9a plots the average RMS power of beam 1 when the load resistance of beam 2 is set arbitrarily to be 8 MΩ. Likewise, the load resistance for beam 1 is chosen arbitrarily as 13.5 MΩ in Figure 9b. For the purpose of the following comparisons, the maximum average RMS power is numerically obtained and denoted by $E_{max}$ in Figure 9. As shown in Figure 9a, when the load resistance of beam 1 is 13.5 MΩ, the average RMS power of beam 1 becomes maximized (6.62 times larger than the unoptimized case with the load resistances of 1 MΩ). In addition, it is observed that the average RMS power outputs evaluated with the optimized and matched resistances are almost the same as shown in the inset of Figure 9a. Both the results are in an excellent agreement with the maximum average RMS result. The optimized average RMS power is improved by 6.56 times relative to the unoptimized case.

In Figure 9b for beam 2, when the load resistance of beam 2 is 8 MΩ, the average RMS power of beam 2 is maximized (3.75 times larger than the unoptimized case). It is noticeable that the average RMS power estimated with the proposed optimization method is still in excellent agreement with the numerically obtained one; on the other hand, the average RMS power estimated with the conventional impedance matching method shows a poor agreement. The average RMS power outputs with the optimized and matched resistances are improved by 3.72 times and 2.83 times, respectively, when compared to the output with the unoptimized resistances. This indicates that it is very important to consider the harmonic distortion in interwell oscillation when designing the load resistance of the 2-DOF MCBEH. The value obtained from Equation (15) at the highest excitation frequency (17.5 Hz in this study) is a good approximation to the optimal load resistance of the 2-DOF MCBEH for broadband energy harvesting applications. Table 2 summarizes the values of resistance and improvements in broadband performance relative to the unoptimized case.

Total average RMS power, a sum of the average RMS powers of two beams, is shown in Figure 9c. The total average RMS power with the optimized resistances (when optimized at 17.5 Hz) is also nearly close to the numerical maximum value. The proposed optimization method can estimate the optimal load resistance for maximizing the total average RMS power more accurately than the conventional impedance matching technique. The total average RMS powers for the optimized and matched cases are improved by 5.28 and 4.88 times, respectively, relative to that of the unoptimized case.

## 5. Conclusions

The optimization method for the load resistances of the 2-DOF MCBEH was proposed in this study when high-order harmonics, i.e., harmonic distortions, emerge due to nonlinear magnetic coupling. First, the 2-DOF oscillator model was obtained through a series of mathematical derivations. The dynamical analysis of the oscillator model was performed by using the direct numerical integration based on the Runge–Kutta method to investigate the nonlinear interwell oscillations of the 2-DOF MCBEH. From the dynamic simulation results, the third-harmonic distortion was found to occur in the interwell oscillation of the shorter beam of the two piezoelectric beams of the 2-DOF MCBEH. Furthermore, the frequency-domain analysis of the steady-state response showed that the third-harmonic distortion became more significant with a higher excitation frequency within the frequency range of interwell motion. Thus, the third-harmonic distortion was considered to be important in the optimization process of the external load resistance in this study. The optimal load resistances for the two piezoelectric beams were estimated by the proposed optimization process. Subsequently, the results of the power outputs were compared to the numerically obtained results as well as the results acquired by employing the conventional impedance matching technique. For both harmonic and swept-sine excitations, the power outputs with the proposed optimal load resistances were in excellent agreement with the numerically optimized power outputs. However, the results obtained by the conventional impedance matching technique were not accurate, and its relative error tended to be larger at a higher excitation frequency where the effect of the harmonic distortion became significant. All the simulation results indicated that the proposed optimization method estimated the optimal load resistances of the 2-DOF MCBEH more accurately than the conventional impedance matching technique did. The proposed optimization method can be readily extended and applied to other multi-DOF systems that experience high-order harmonic distortions.

## Figures and Tables

**Table 1:** Geometric dimensions and material properties of the 2-DOF MCBEH. The values in the parentheses are the values for beam 2.

	Metal Substrate	Piezoelectric Layer	Magnet
Length	73 mm (44 mm)	73 mm (44 mm)	2 mm
Thickness	0.3 mm	0.052 mm	6 mm
Width	10 mm	10 mm	10 mm
Density	7850 kg/m^3^	1780 kg/m^3^	-
Young’s modulus	200 GPa	3 GPa	-
Piezoelectric strain constant	-	−23 pm/V	-
Permittivity	-	110 pF/m	-
Mass	-	-	1 g
Mass moment of inertia	-	-	4 g·mm^2^
Magnetization	-	-	900 kA/m
Separation distance	-	-	11.6 mm

**Table 2:** Summary of the values of the external load resistance and relative improvements in broadband performance.

Method	Beam 1 (*Negligible Harmonic Distortion*)		Beam 2 (*Noticeable Third-Harmonic Distortion*)	
	Resistance (MΩ)	Improvement	Resistance (MΩ)	Improvement
Unoptimized case	1.0	-	1.0	-
Conventional impedance matching	12.0	6.56 times	19.8	2.83 times
Proposed optimization method	11.9	6.56 times	6.8	3.72 times
Numerically obtained maximum	13.5	6.62 times	8.0	3.75 times

## References

[1] Chen X., Xu S., Yao N., Shi Y. (2010). 1.6 V Nanogenerator for Mechanical Energy Harvesting Using PZT Nanofibers. Nano Lett..

[2] Bakytbekov A., Nguyen T.Q., Huynh C., Salama K.N., Shamim A. (2018). Fully printed 3D cube-shaped multiband fractal rectenna for ambient RF energy harvesting. Nano Energy.

[3] Kishore R.A., Priya S. (2018). A review on low-grade thermal energy harvesting: Materials, methods and devices. Materials.

[4] Wang J., Geng L., Ding L., Zhu H., Yurchenko D. (2020). The state-of-the-art review on energy harvesting from flow-induced vibrations. Appl. Energy.

[5] Lechêne B.P., Cowell M., Pierre A., Evans J.W., Wright P.K., Arias A.C. (2016). Organic solar cells and fully printed super-capacitors optimized for indoor light energy harvesting. Nano Energy.

[6] Lv J., Jeerapan I., Tehrani F., Yin L., Silva-Lopez C.A., Jang J.-H., Joshuia D., Shah R., Liang Y., Xie L. (2018). Sweat-based wearable energy harvesting-storage hybrid textile devices. Energy Environ. Sci..

[7] Harb A. (2011). Energy harvesting: State-of-the-art. Renew. Energy.

[8] Tan T., Yan Z., Zou H., Ma K., Liu F., Zhao L., Peng Z., Zhang W. (2019). Renewable energy harvesting and absorbing via multi-scale metamaterial systems for Internet of things. Appl. Energy.

[9] Yang Z., Zhou S., Zu J., Inman D. (2018). High-performance piezoelectric energy harvesters and their applications. Joule.

[10] Vullers R.J., Van Schaijk R.R., Visser H.J., Penders J., Van Hoof C. (2010). Energy Harvesting for Autonomous Wireless Sensor Networks. IEEE Solid-State Circuits Mag..

[11] Liu X., Zhang X. (2018). Rate and energy efficiency improvements for 5G-based IoT with simultaneous transfer. IEEE Internet Things J..

[12] Zhang Y., Xie M., Adamaki V., Khanbareh H., Bowen C.R. (2017). Control of electro-chemical processes using energy harvesting materials and devices. Chem. Soc. Rev..

[13] Núñez C.G., Manjakkal L., Dahiya R. (2019). Energy autonomous electronic skin. npj Flex. Electron..

[14] Jiang D., Shi B., Ouyang H., Fan Y., Wang Z.L., Li Z. (2020). Emerging Implantable Energy Harvesters and Self-Powered Implantable Medical Electronics. ACS Nano.

[15] Cook-Chennault K.A., Thambi N., Sastry A.M. (2008). Powering MEMS portable devices—A review of non-regenerative and regenerative power supply systems with special emphasis on piezoelectric energy harvesting systems. Smart Mater. Struct..

[16] Tran N., Ghayesh M.H., Arjomandi M. (2018). Ambient vibration energy harvesters: A review on nonlinear techniques for performance enhancement. Int. J. Eng. Sci..

[17] Daqaq M.F., Masana R., Erturk A., Dane Quinn D. (2014). On the role of nonlinearities in vibratory energy harvesting: A critical review and discussion. Appl. Mech. Rev..

[18] Szemplińska-Stupnicka W., Rudowski J. (1993). Steady states in the twin-well potential oscillator: Computer simulations and approximate analytical studies. Chaos.

[19] Fu H., Yeatman E.M. Broadband rotational energy harvesting using bistable mechanism and frequency up-conversion. Proceedings of the 2017 IEEE 30th International Conference on Micro Electro Mechanical Systems (MEMS).

[20] Nguyen M.S., Yoon Y.-J., Kwon O., Kim P. (2017). Lowering the potential barrier of a bistable energy harvester with mechanically rectified motion of an auxiliary magnet oscillator. Appl. Phys. Lett..

[21] Nguyen M.S., Yoon Y.-J., Kim P. (2019). Enhanced broadband performance of magnetically coupled 2-DOF bistable energy harvester with secondary intrawell resonances. Int. J. Precis. Eng. Man. Technol..

[22] Kim H., Priya S., Stephanou H., Uchino K. (2007). Consideration of impedance matching techniques for efficient piezoelectric energy harvesting. IEEE Trans. Ultrason. Ferroelectr. Freq. Control.

[23] Song H.-C., Kumar P., Sriramdas R., Lee H., Sharpes N., Kang M.-G., Maurya D., Sanghadasa M., Kang H.-W., Ryu J. (2018). Broadband dual phase energy harvester: Vibration and magnetic field. Appl. Energy.

[24] Liu W., Badel A., Formosa F., Wu Y., Agbossou A. (2013). Novel piezoelectric bistable oscillator architecture for wideband vibration energy harvesting. Smart Mater. Struct..

[25] Liang J., Liao W.-H. (2010). Impedance matching for improving piezoelectric energy harvesting systems. Proc. Act. Passiv. Smart Struct. Integr. Syst..

[26] Bae S., Kim P. (2021). Load Resistance Optimization of a Broadband Bistable Piezoelectric Energy Harvester for Primary Harmonic and Subharmonic Behaviors. Sustainability.

[27] Bae S., Kim P. (2021). Load Resistance Optimization of Bi-Stable Electromagnetic Energy Harvester Based on Harmonic Balance. Sensors.

[28] Allane D., Vera G.A., Duroc Y., Touhami R., Tedjini S. (2016). Harmonic power harvesting system for passive RFID sensor tags. IEEE Trans. Microw. Theory Tech..

[29] Vera G.A., Duroc Y., Tedjini S. (2015). Third harmonic exploitation in passive UHF RFID. IEEE Trans. Microw. Theory Tech..

[30] Erturk A., Inman D.J. (2011). Piezoelectric Energy Harvesting.

[31] Kim P., Nguyen M.S., Kwon O., Kim Y.-J., Yoon Y.-J. (2016). Phase-dependent dynamic potential of magnetically coupled two-degree-of-freedom bistable energy harvester. Sci. Rep..

[32] Deng H., Du Y., Wang Z., Ye J., Zhang J., Ma M., Zhong X. (2019). Poly-stable energy harvesting based on synergetic multistable vibration. Commun. Phys..

